# Integrated analysis of the molecular pathogenesis of FDXR-associated disease

**DOI:** 10.1038/s41419-020-2637-3

**Published:** 2020-06-04

**Authors:** Jesse D. Slone, Li Yang, Yanyan Peng, Luis F. Queme, Belinda Harris, Stacey J. Sukoff Rizzo, Torrian Green, Jennifer L. Ryan, Michael P. Jankowski, Laura G. Reinholdt, Taosheng Huang

**Affiliations:** 1 Division of Human Genetics, Cincinnati Children’s Hospital Medical Center, Cincinnati, OH USA 45229; 2 Division of Anesthesia, Cincinnati Children’s Hospital Medical Center, Cincinnati, OH USA 45229; 3 The Jackson Laboratory, Bar Harbor, ME USA 04660; 4 University of Pittsburgh School of Medicine, Pittsburgh, PA USA 15219

**Keywords:** Mitochondria, Neurodegeneration

## Abstract

The mitochondrial flavoprotein ferredoxin reductase (FDXR) is required for biogenesis of iron–sulfur clusters and for steroidogenesis. Iron–sulfur (Fe–S) clusters are ubiquitous cofactors essential to various cellular processes, and an increasing number of disorders are associated with disruptions in the synthesis of Fe–S clusters. Our previous studies have demonstrated that hypomorphic mutations in FDXR cause a novel mitochondriopathy and optic atrophy in humans and mice, attributed in part to reduced function of the electron transport chain (ETC) as well as elevated production of reactive oxygen species (ROS). Inflammation and peripheral neuropathy are also hallmarks of this disease. In this paper, we demonstrate that *FDXR* mutation leads to significant optic transport defects that are likely to underlie optic atrophy, a major clinical presentation in FDXR patients, as well as a neurodegenerative loss of cells in the central nervous system (CNS). Molecular analysis indicates that FDXR mutation also leads to mitochondrial iron overload and an associated depolarization of the mitochondrial membrane, further supporting the hypothesis that FDXR mutations cause neurodegeneration by affecting FDXR’s critical role in iron homeostasis.

## Introduction

Iron overload is a frequently observed occurrence in many mitochondrial diseases, as well as a common cause for neurological disorders. Iron is essential for many cellular and biochemical activities, including energy production and biogenesis of iron–sulfur (Fe–S) clusters^1^, which are complexed into a variety of proteins as enzymatic cofactors. Improper synthesis of these Fe–S clusters can lead to mislocalization or excessive deposits of iron within tissues and cells, leading to pathological changes and cellular dysfunction. A number of disorders have been associated with the loss of Fe–S clusters and the resulting accumulation of iron. As an example, Friedreich’s Ataxia—one of the most common forms of inherited hereditary ataxia—is caused by the unstable expansion of a GAA trinucleotide repeat sequence within the frataxin (*FXN*) gene. *FXN* encodes a mitochondrial protein critical to the production of Fe–S clusters within the mitochondrion^2–4^. Although the mechanism by which the FXN protein synthesizes Fe–S clusters is poorly understood, loss-of-function mutations result in elevated iron levels within the mitochondria and mitochondrial dysfunction^5^. This, in turn, leads to the onset of a host of symptoms (usually before the age of 20), including ataxia^6^, cardiomyopathy, and neurodegeneration in the cerebellum^7^. There is also evidence that elevated iron levels may be at least partially responsible for the damage to dopaminergic neurons that occurs in Parkinson’s disease^8^. Finally, there is an entire class of rare, inherited neurodegenerative diseases referred to as NBIA (neurodegeneration with brain iron accumulation), which are characterized by a progressive neurodegeneration phenotype linked with an abnormal and distinct accumulation of iron in the basal ganglia^9–12^. Together, all of these lines of evidence suggest that dysregulated iron metabolism may be a recurring theme in neurodegenerative disorders.

Ferredoxin reductase (FDXR) is a mitochondrial membrane-associated flavoprotein. One of its functions is to transfer electrons from NADPH to the two human ferredoxin proteins, FDX1 and FDX2^13,14^. FDX1, in turn, reduces cytochrome P450, giving FDXR an indirect but critical role in steroid biosynthesis and drug metabolism pathways. Interestingly, FDX2 appears to be the more critical of the two ferredoxins in the iron–sulfur pathway, as deletion of FDX1 in HeLa cells does not elicit any defects in Fe–S cluster biogenesis^14^, while mutation of FDX2 has been associated with a mitochondrial muscle myopathy and severely impaired activity of Fe–S proteins in mitochondria^15^. Furthermore, while FDX1 is exclusively expressed in adrenal gland, FDX2 is ubiquitously found in virtually all tissues^14^. Thus, FDX1 (and indirectly, FDXR) is likely to participate in the synthesis of bile acid, vitamin D, and steroid hormones^13,16–18^, while FDX2 is likely involved in Fe–S cluster biogenesis.

Having established the role of FDXR in Fe–S cluster assembly, it is perhaps unsurprising to learn that knockdown of FDXR activity leads to mitochondrial iron overload in cell culture^13^. However, despite such tantalizing clues and a well-defined biochemical function, the exact consequences of FDXR mutation in patients have remained largely elusive. Using data from a large number of families, we recently demonstrated that biallelic mutations in the *FDXR* gene lead to gait abnormalities and visual impairment^19^. In a parallel analysis, we showed that mice possessing a naturally occurring mutation in *Fdxr* presented with neurological abnormalities, loss of visual acuity and gait abnormalities, reminiscent of the phenotypes observed in patients. Enzymatic assays in both patient fibroblasts and in tissues from *Fdxr* mutant mice indicated multiple biochemical and metabolic consequences of FDXR mutation, including reductions in ATP production and ETC complex activities, along with a significant increase in the production of reactive oxygen species (ROS)^19^. Furthermore, our follow-up study showed that FDXR mutation is linked to inflammation and gliosis in the CNS, providing clues as to the possible mechanism of FDXR pathology^20^. Finally, an independent study published by a separate group has reported eight individuals in four different families carrying loss-of-function mutations in FDXR who also presented with mitochondriopathy and peripheral sensory neuropathies in the auditory and optic systems^21^, providing further corroboration of the neurodegenerative consequences of FDXR mutation.

However, the mechanism by which FDXR mutation leads to the observed neuropathy and optic atrophy is largely unknown. Based on studies of Friedreich’s Ataxia and other conditions in the iron–sulfur cluster synthesis pathway, iron overload is a likely mechanism of FDXR pathogenesis. Here, we show that abnormal iron accumulation is indeed prevalent in *FDXR* patient fibroblasts and in the tissues of *Fdxr*^R389Q/R389Q^ mice, leading to molecular and cellular dysfunctions such as reduced mitochondrial membrane potential and optic transport defects. Together, these results provide a better understanding of how loss of FDXR function leads to the novel mitochondriopathy associated with FDXR dysfunction and opens a potential avenue to treat this group of patients.

## Results

### Fdxr mutations cause a progressive optic neuropathy and optic transport defects in *Fdxr*^*−/*−^ mice

**Fig. 1:**
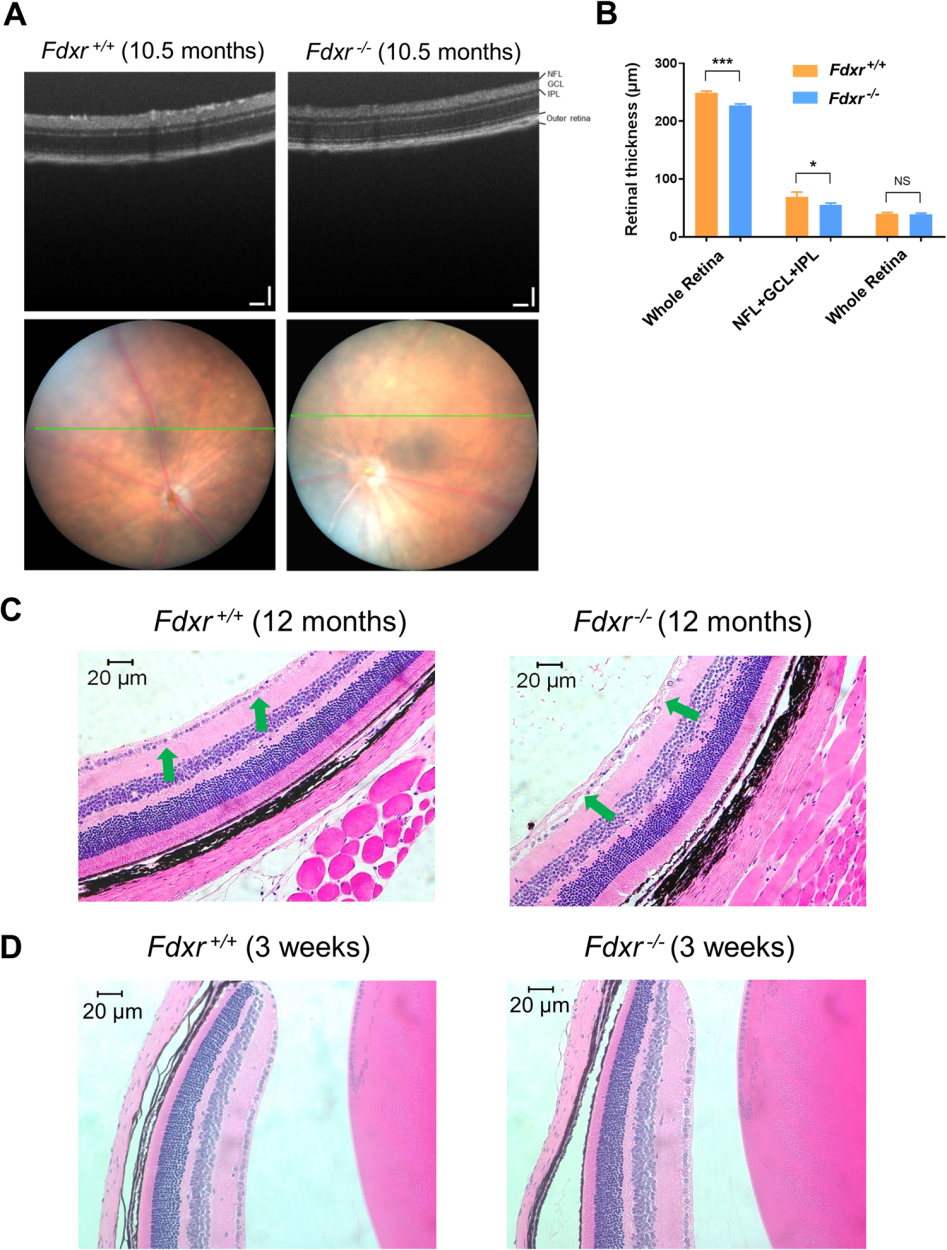
*Fdxr*-mediated optic atrophy and RGC loss. **a** Optic disc and OCT scanning of P120 mice. The green line in optic disc images indicates the position of OCT scanning. Scale bars: 100 μm. **b** Quantification of retinal thickness from OCT scanning images of retinas from *Fdxr*^+*/*+^ and *Fdxr*^−/−^ mice (*P < 0.05, **P < 0.01, ***P < 0.001); *n* = 14 retinal images derived from two animals, per genotype). **c** H&E-stained sagittal sections through the optic disc show that the number of retinal ganglion cells (RGCs) in the Ganglion Cell Layer (green arrows) are markedly reduced in H&E sections of eyes from 12-month-old *Fdxr*^−*/−*^ mutant mice, as compared with *Fdxr*^+*/*+^ control mice. **d** H&E staining of retinal sections from mice at 3 weeks of age indicates no difference in number of RGCs between *Fdxr*^+*/*+^ and *Fdxr*^−*/−*^ animals.

The naturally occurring *Fdxr*^R389Q/R389Q^ mouse line^22^ harbors a homozygous R389Q missense mutation in *Fdxr* that is allelic to the common human mutation p.R392W^19^. To better characterize the optic neuropathy associated with FDXR mutation, we examined the retina of *Fdxr*^R389Q/R389Q^ mutant mice (hereafter referred to as “*Fdxr*^−/−^” mice) with an imaging technique, optical coherence tomography (OCT), that can acquire cross-sectional tomographic images of the retina in vivo^23,24^. Although the optic disc appeared grossly normal under bright-field examination, OCT scanning revealed significantly thinner retinas in aged *Fdxr*^−/−^ mice (Fig. 1a). Quantitative measurements indicated that the average thickness of the whole retina and ganglion cell complex (GCC)—which includes the nerve fiber layer (NFL), ganglion cell layer (GCL), and inner plexiform layer (IPL)—were also decreased in *Fdxr*^−/−^ compared with *Fdxr*^+/+^ control mice at 10.5 months (Fig. 1b). In contrast, outer retinal thickness was relatively normal.

To further extend this analysis, we performed a histopathological assessment of the retina. We counted the retinal ganglion cell (RGC) layer nuclei in H&E-stained paraffin sections of eyes from 3-week-old and 1-year-old *Fdxr*^+/+^ and *Fdxr*^−/−^ mice. Retinal H&E-stained sections displayed normal organization of retinal structure, but thinner inner retinas and noticeable retinal ganglion cell (RGC) loss in the GCL at 12 months (Fig. 1c), in agreement with our previous data^19^. By contrast, there was no significant difference in the number of RGC layer nuclei between *Fdxr*^+/+^ and *Fdxr*^−/−^ mice at 3 weeks (Fig. 1d), suggesting RGC loss is a progressive, age-dependent process.

**Fig. 2:**
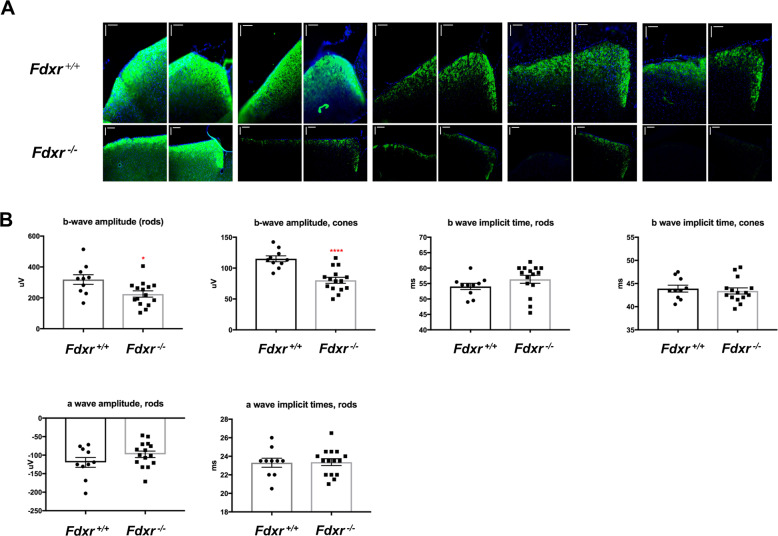
*Fdxr* mutation causes functional defects in retinal neurons. **a** Anterograde axonal transport of mouse retinal ganglion cells (RGCs) was measured in the mouse eyes. Seven-month-old *Fdxr*^+*/*+^ control and *Fdxr*^*−/−*^ mutant mice were anesthetized with an inhalation of 2.5% isoflurane. Brains were cryo-sectioned through the superior colliculi with a thickness of 50 µm. Transport of CTB-Fluor 488 from the eye to the superior colliculi in the brain was greatly reduced in the *Fdxr*^*−/−*^ mutant mice, as compared with the transport in the *Fdxr*^+*/*+^ control mice. Scale bars: 100 μm. **b** Electroretinography was performed in 4-month-old *Fdxr*^+*/*+^ and *Fdxr*^*−/−*^ mouse eyes. After light stimulation, both ocular photoreceptor rods and cones showed decreased b-wave amplitude in the *Fdxr*^−*/*−^ mutants relative to control mice.

Given the mounting evidence that *FDXR* mutation causes optic atrophy and loss of retinal cells^19^, it is reasonable to examine anterograde axonal transport within mouse RGCs. In this experiment, cholera toxin subunit B (CTB) conjugated with Alex-Fluor 488 was injected into the eyes of 7-month-old *Fdxr*^+/+^ and *Fdxr*^−/−^ mice. Two days after injection, the brains were harvested, fixed, and cryo-sectioned through the superior colliculi. As shown in Fig. 2a, transport of the CTB-Fluor 488 marker from the eye to the superior colliculi in the brain was greatly reduced in the *Fdxr*^−/−^ mutant mice, as compared with their age-matched *Fdxr*^+/+^ controls. Electroretinography was also performed separately on the eyes of 4-month-old *Fdxr*^+/+^ and *Fdxr*^−/−^ mice. The results demonstrated that both ocular photoreceptor rods and cones showed decreased b-wave amplitude in response to light stimulation (Fig. 2b), indicating that mutant retinal cells are functionally compromised.

### *Fdxr* mutation leads to an age-dependent loss of cells in the CNS

**Fig. 3:**
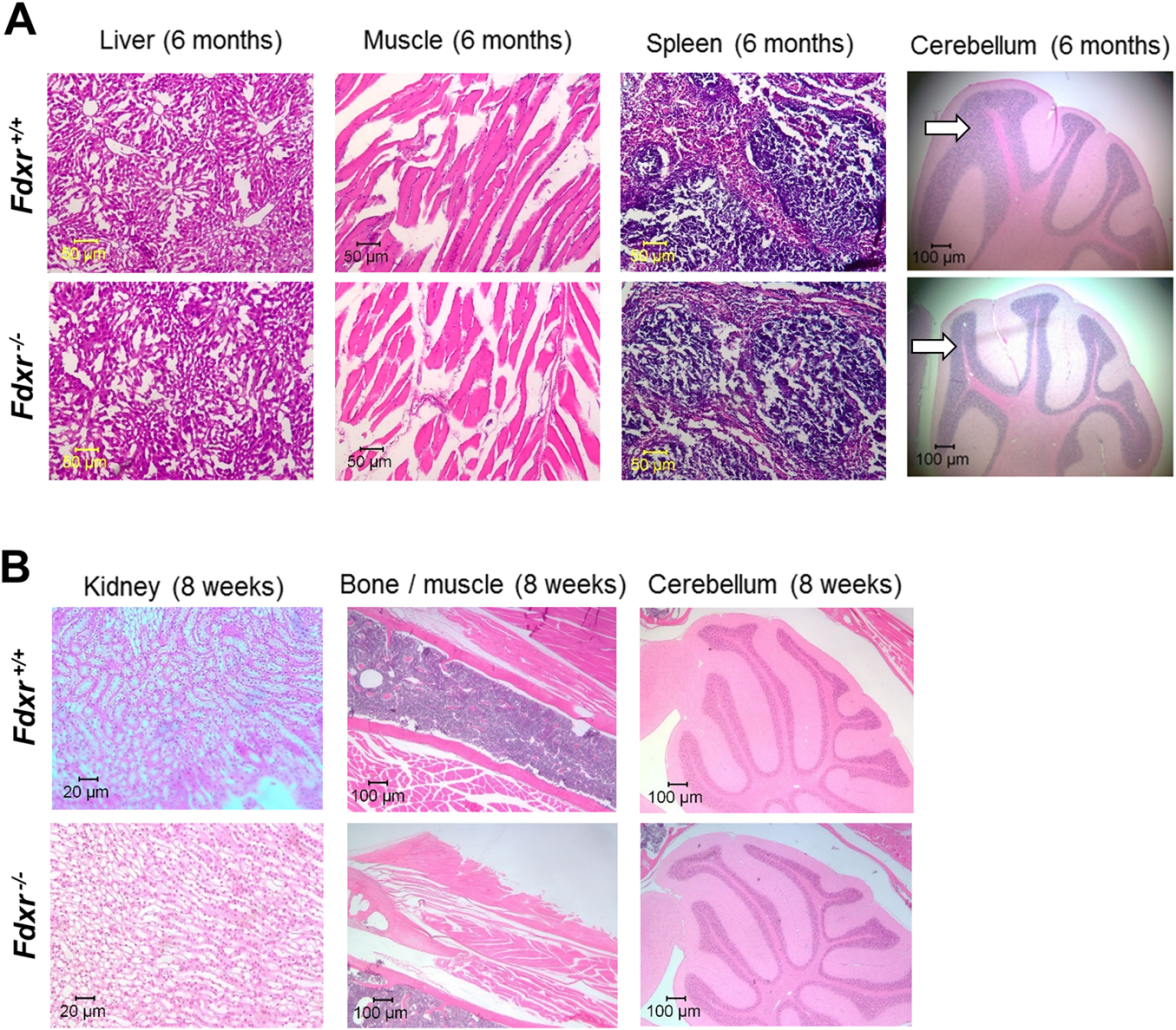
Pathology assessment of tissues from Fdxr mutant mice. **a** H&E staining of *Fdxr* mutant mouse tissues from the liver, muscle, spleen, and brain/cerebellum from 6-month-old *Fdxr*^*−/−*^ mice as compared with *Fdxr*^*+/+*^ control mice. The cerebellar granular layers for the mutant and control samples are indicated with white arrows. In general, the cerebellar granular layer of the mutant sample shows a reduced volume (relative to the other cerebellar layers) than what is observed in the control sample. **b** H&E staining of tissues from the kidneys, bone, and muscle, and cerebellum from 2-month-old *Fdxr*^−***/***−^ mice as compared with *Fdxr*^*+/+*^ control mice.

To more closely examine the neurodegenerative effect of *Fdxr* mutation in the mouse model, H&E staining was performed on tissues from the liver, muscle, spleen, and brain/cerebellum of 6-month-old *Fdxr*^*−/−*^ mice and compared with tissues from age-matched *Fdxr*^+/+^ controls (Fig. 3a). As an age comparison, H&E staining was also performed on the kidney, bone, muscle, and cerebellum tissue from 2-month-old *Fdxr*^−*/*−^ mice and from age-matched *Fdxr*^+/+^ controls (Fig. 3b), as well as from the occipital lobe (Supplementary Fig. S1B) and the spinal cord (Supplementary Fig. S1A). In general, the most dramatic cell reduction was observed in the mutant CNS samples, particularly in the cerebellar granular layer of 6-month-old mice (Fig. 3a, white arrows). This cell loss is likely related to the neurodegenerative effects that we have demonstrated previously^19,20^. Examination of tissue sections from the occipital lobe also revealed a reduction in the number of cells in the visual cortex of the *Fdxr*^−*/−*^ mutants even at 8 weeks of age (Supplementary Fig. S1B), which may further compound the optic atrophy that we have previously observed^19^.

### *Fdxr* mutation causes peripheral neuropathy associated with axon degeneration and movement defects

**Fig. 4:**
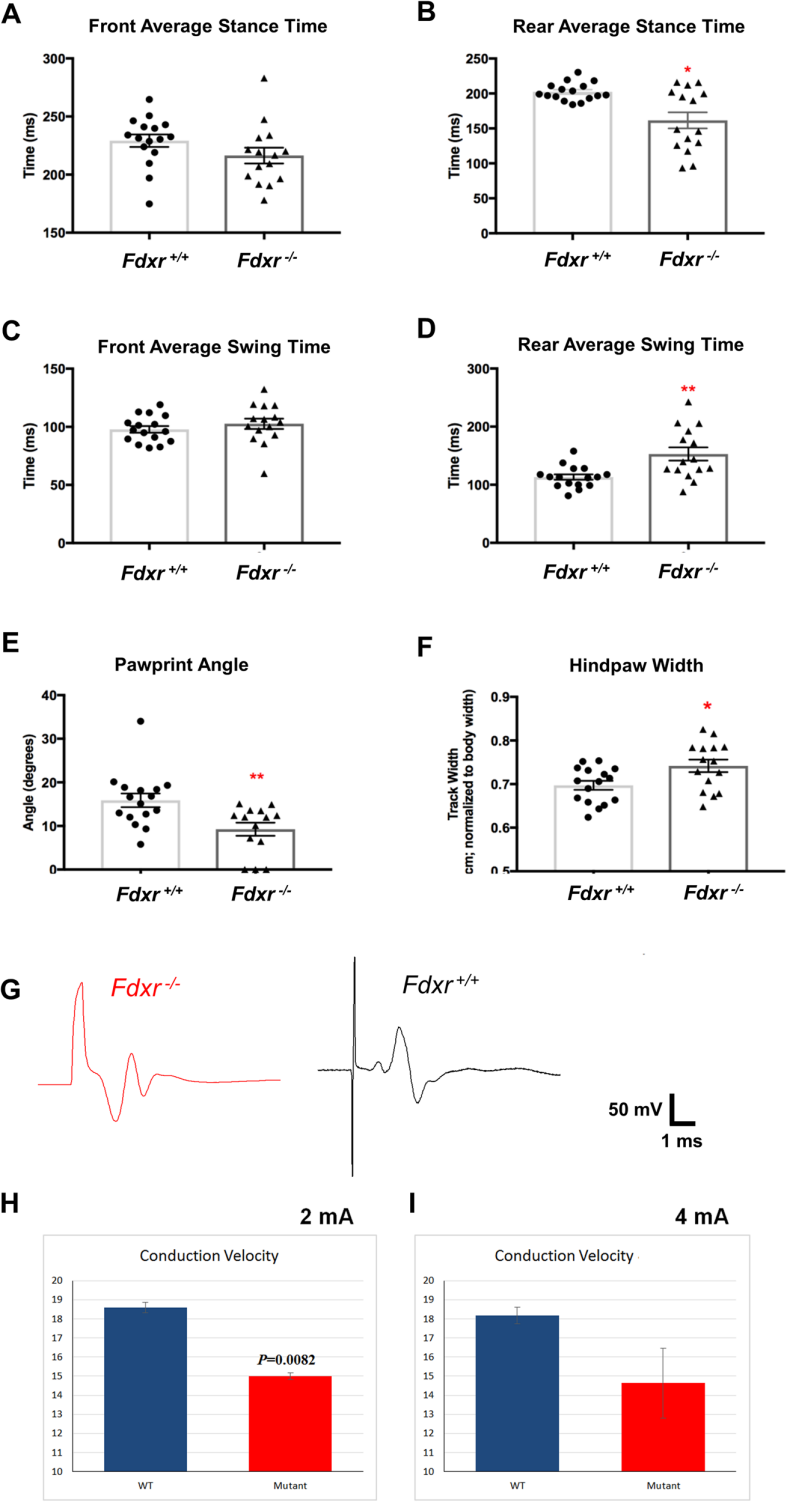
Gait abnormalities and peripheral nerve conduction defects of *Fdxr*^*R389Q/R389Q*^ mutant mice relative to wild-type (WT) mice. Significant impairments in rear-gait dynamics (slower swing times and shorter time in stance position) were observed in *Fdxr* mutants relative to WT controls independent of sex (**b**, **d**), whereas front-gait dynamics appeared unaffected (**a**, **c**). Altered rear-gait dynamics in *Fdxr*^*R389Q/R389Q*^ were associated with abnormal hindpaw position and significantly impaired sciatic function as measured by pawprint angle (**e**) and impaired hindlimb base support as measured by increased hindpaw width (**f**). To confirm the underlying neurophysiology of these behavioral defects, CMAP recordings were taken from sciatic nerves of *Fdxr*^*+/+*^ (black) and *Fdxr*^*−/−*^ (red) mice at 2 months of age (**g**). Arrows indicate onset of electrical stimulation of the sciatic nerve during recording. Conduction velocities (CVs) were measured from recorded waves using a 2 mA (**h**) or 4 mA (**i**) electrical stimulation of the sciatic nerve (*n* = 16 for *Fdxr*^*+/+*^ and *Fdxr*^−*/−*^ mice; *P* < 0.001). Data are representative of two independent experiments.

Homozygous *Fdxr*^−/−^ mice demonstrate stiffness in their hindquarters by 7–8 weeks of age that progresses to a severe reduction in ambulation (Fig. 4a–f), similar to the phenotype observed in patients carrying the p.R392W mutation. To more fully quantify the altered movement behavior of these mice, we conducted comprehensive gait analyses using a standard treadmill system. At 4–9 weeks of age, significant differences in rear-gait measurements— including impaired gait dynamics (swing and stance) (Fig. 4a–d), rear sciatic functional impairments as measured by the pawprint angle (Fig. 4e), and altered hindlimb base support as measured by increased hindpaw width (Fig. 4f)—were observed across both sexes in *Fdxr*^−/−^ homozygous mice relative to age- and sex-matched *Fdxr*^+/+^ controls. By 10–13 weeks of age, advanced gait impairment prevented most *Fdxr*^−/−^ homozygous mice from performing these tests, confirming that *Fdxr* mutation leads to severe defects in the control of movement behavior.

Six-month-old *Fdxr*^−/−^ mice also display enhanced hindlimb-clasping reflex and muscle atrophy, indicative of potential peripheral neuropathy (Supplementary Figs. S2A and S2B). In many human peripheral neuropathies, degeneration is associated with muscle atrophy and lower extremity dysfunction. We examined peripheral nerve conduction in *Fdxr*^*−/−*^ mice by acquiring compound muscle action potentials (CMAPs) from the lateral gastrocnemius muscle during direct electrical stimulation of the exposed sciatic nerve in vivo. Based on recorded electromyography (EMG) waves (Fig. 4g), conduction velocity (CV) was decreased in *Fdxr*^*−/−*^ mice at 6 months compared with normal littermates (Fig. 4h, i), suggesting that myelination defects may underlie this aspect of *Fdxr* pathology.

Interestingly, our results also showed an observable increase in the number of cells in the spinal cord sections of eight-week old *Fdxr*^*−/−*^ mutant mice (Supplementary Fig. S1A), as well as a significant increase in the cell number in the superficial area (Supplementary Fig. S1C), middle area (Supplementary Fig. S1D), and deeper area (Supplementary Fig. S1E) of the cerebral cortex. Given the clear reduction in cell number observed in mutant CNS tissue by 6 months of age (Fig. 3a), it is likely that the increased cell number observed in 8-week-old mutant mice represent peripheral immune cells that have been recruited as a result of the neurodegenerative process itself. This phenomenon has been fairly well-documented in the literature^25^, and would not be expected to stave off neuronal cell loss in the long term. Thus, the initial increase in cell number would not be expected to be maintained with age, leading to the dramatic loss of CNS cells observed in older mouse tissues.

### *Fdxr* mutation disrupts iron metabolism, leading to iron accumulation

**Fig. 5:**
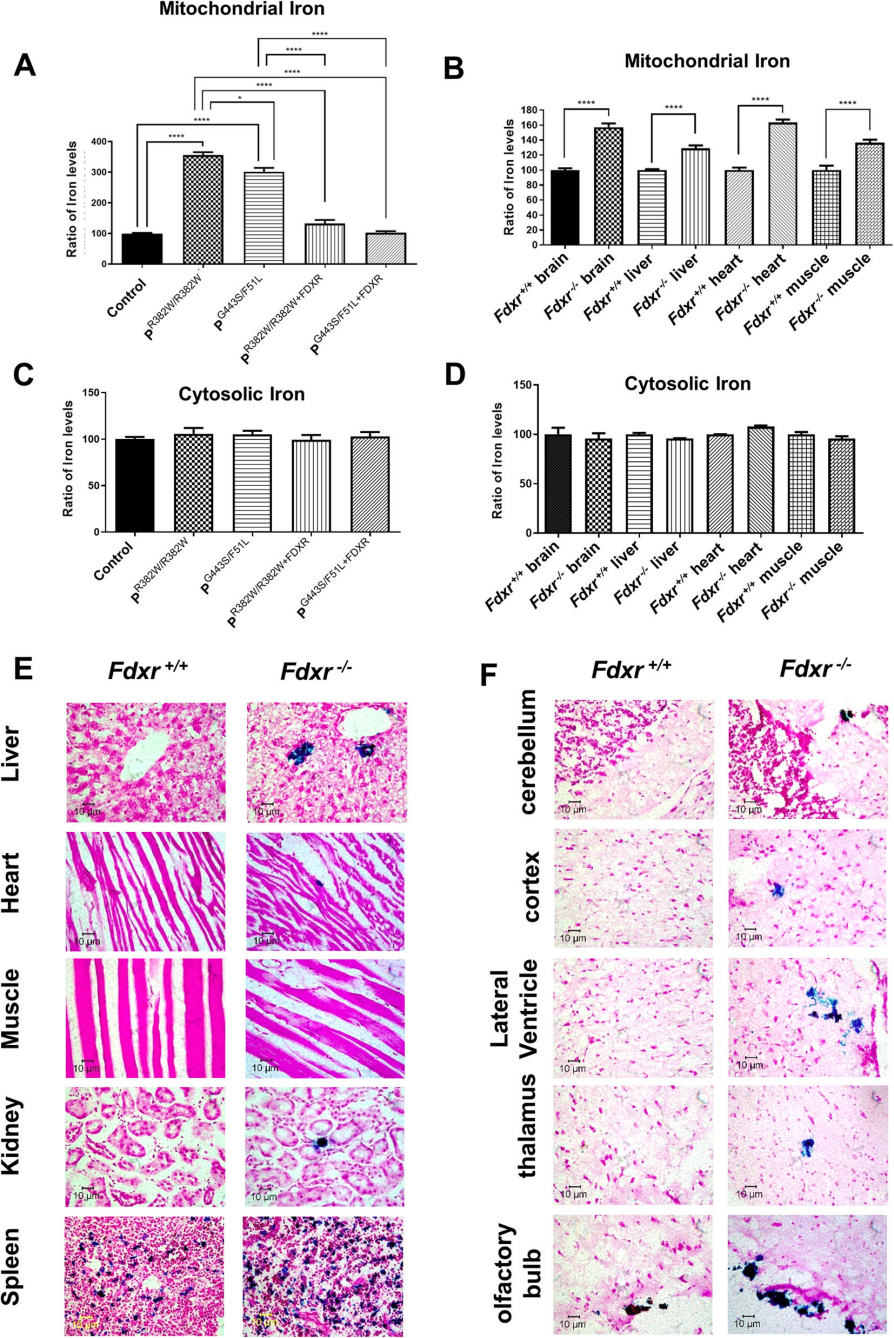
*FDXR* mutation regulates iron metabolism. Iron levels (Fe^2+^ plus Fe^3+^) were measured by QuantiChrom iron assay in extracts from control and patient fibroblasts carrying *FDXR*^*R392W****/****R392W*^ or *FDXR*^*G443S/F51L*^ mutations (**a**, **c**), and in extracts from *Fdxr*^−/−^ mutant mice and *Fdxr*^+*/*+^ littermates (**b**, **d**). Iron levels in mitochondrial extracts (**a**, **b**) were reduced in *FDXR* mutant cells and tissues. In contrast, cytoplasmic extracts showed no significant differences between *FDXR* mutant cells/tissues and their corresponding controls (**c**, **d**). **e** Fdxr deficiency leads to iron overload at the age of 10.5 months in mice. Tissues from *Fdxr*^−*/−*^ mutant mice and *Fdxr*^+*/*+^ littermates were stained with Prussian blue to measure iron accumulation. The results are shown for the liver, heart, muscle, kidney, and spleen. **f** Prussian blue staining was also carried on various brain tissues from *Fdxr*^−/−^ and *Fdxr*^+*/*+^ mice. The results show increased iron levels in the cerebellum, cortex, lateral ventricle, thalamus, and olfactory bulb.

Despite its well-characterized role in iron metabolic pathways, the in vivo role of FDXR in iron metabolism remains relatively unexplored. To test the hypothesis that *FDXR* mutation modulates iron metabolism, total cellular iron and iron in the mitochondrial fraction were measured using the QuantiChrom iron assay. In patient fibroblasts, we found that FDXR mutations lead to a dramatic accumulation of iron in mitochondrial-specific extracts (Fig. 5a). This effect was recapitulated in the mouse model (Fig. 5b), where we also demonstrated that *Fdxr* mutation causes iron accumulation in mitochondrial extracts from multiple tissues (including the brain, liver, heart, and muscles). Iron accumulation in the mouse model was further confirmed by Prussian blue staining of *Fdxr*^*−/−*^ mouse tissues (at the age of 10.5 months) (Fig. 5e, f). Interestingly, the QuantiChrom assay showed that cytosolic extracts as a whole possess no discernible difference in iron levels between *Fdxr*^*−/*−^ mutant and *Fdxr*^+*/*+^ control samples (Fig. 5c, d). Taken together, these results conclusively demonstrate that FDXR dysfunction leads to mitochondria-specific iron accumulation across multiple organ systems.

### *Fdxr* mutations compromise the mitochondrial membrane potential

**Fig. 6:**
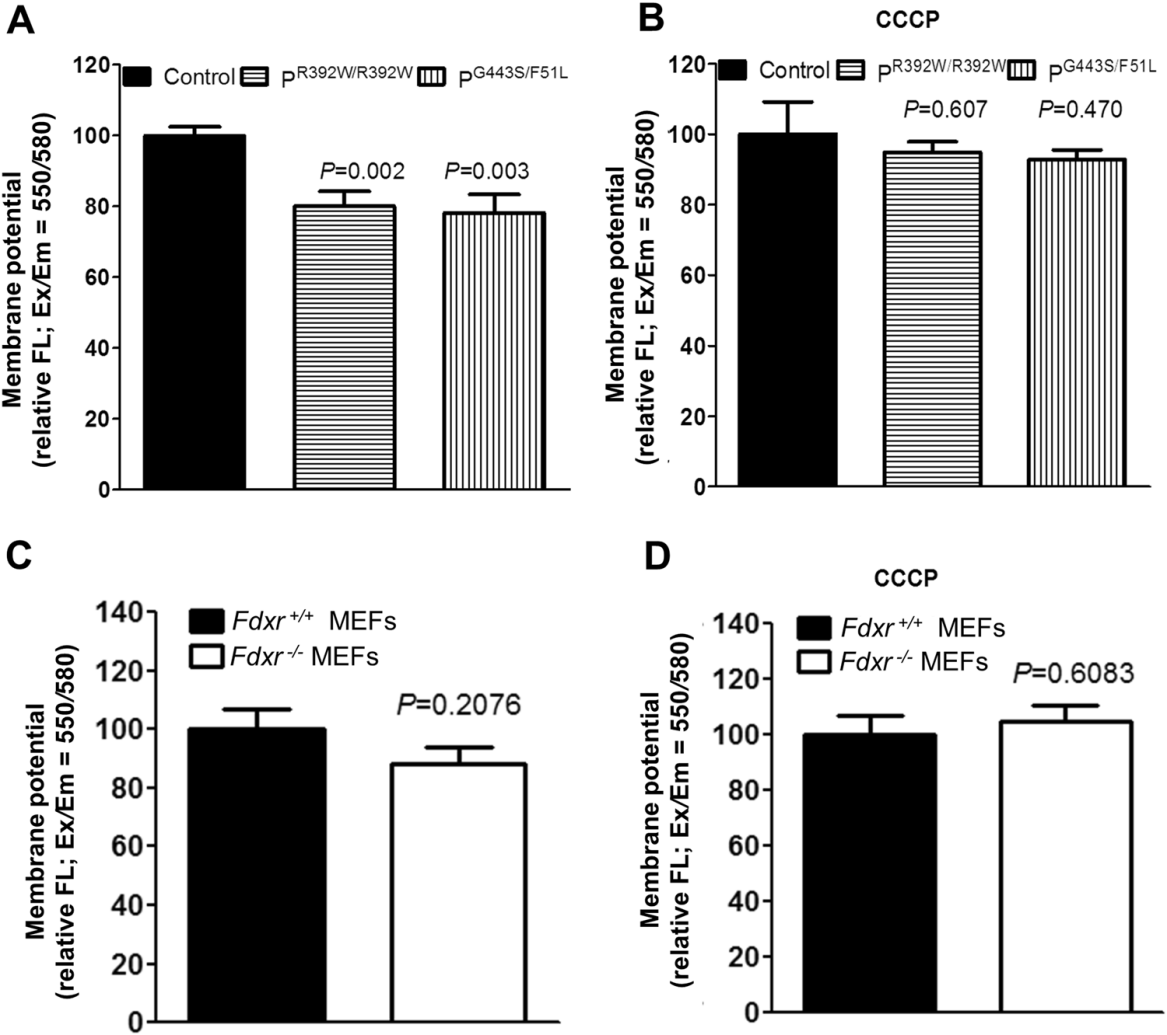
*FDXR* mutation leads to a reduction in mitochondrial membrane potential. **a** The mitochondrial membrane potential (Δ*Ψ*m) was measured in patient fibroblasts with *FDXR*^*R392W****/****R392W*^ or *FDXR*^*G443S/F51L*^ mutations and control fibroblasts, using a TMRE-based assay system. Δ*Ψ*m was determined according to the relative fluorescence intensity at Ex*/*Em = 550*/*580, in the absence of 10 µM of carbonyl cyanide 3-chlorophenylhydrazone (CCCP). **b** Mitochondrial membrane potential of the cell lines from panel **a**, as measured in the presence of 10 µM of carbonyl cyanide 3-chlorophenylhydrazone (CCCP), is shown as a control. **c** Mitochondrial membrane potential, as determined in mouse embryonic fibroblasts (MEFs) from *Fdxr*^*−/−*^ mutant mice and *Fdxr*^+*/*+^ control littermates. **d** Mitochondrial membrane potential of the cell lines from panel **d**, as measured in the presence of 10 µM of CCCP, is shown as a positive control. The average of three to five determinations is shown for each cell line.

The role of iron accumulation in mitochondria associated with FDXR mutations is not fully understood. Previously, mutations of FDXR were associated with multiple biochemical and metabolic abnormalities, including reductions in ATP production, ETC complex activities, and oxygen consumption rates, along with a significant increase in the production of ROS^19^.The deficiency in some complexes could be explained by abnormal iron–sulfur synthesis, as iron–sulfur is the cofactor for those complexes. In support of this model, frataxin mutation has been shown to cause an increase in ROS production, as well as a decrease in mitochondrial membrane potential (Δ*Ψ*m) in Friedreich’s ataxia model^26^. Iron accumulation may also play a more direct role in the process of mitochondrial dysfunction, as iron overload has been reported to directly induce mitochondrial membrane depolarization and increased ROS production in mitochondria isolated from rat cardiac cells^27^. To test whether the iron accumulation in our animal model is associated with changes in mitochondrial membrane potential, Δ*Ψ*m was measured in our patient fibroblasts and *Fdxr*^*−/−*^ mouse embryonic fibroblasts using the cell-permeable fluorescence probe TMRE (tetramethylrhodamine, ethyl ester), which accumulates in mitochondria as a result of their high membrane potential. The fluorescence intensity at Ex*/*Em = 550*/*580 was recorded to delineate the relative Δ*Ψ*m level of mutant and control cell lines. As shown in Fig. 6a, c, the Δ*Ψ*m in the mutant cell lines from patients (Fig. 6a) and the *Fdxr*^*−/−*^ MEF cells (Fig. 6c) were consistently ~15–20% lower than the Δ*Ψ*m observed in their respective control cell lines. In contrast, the Δ*Ψ*m in mutant and patient cells in the presence of CCCP (carbonyl cyanide 3-chlorophenylhydrazone) were comparable with those measured in the control cell lines (Fig. 6b, d). Together, these results support the idea that the accumulation of iron associated with loss of FDXR affects the normal function of mitochondria and comprises the ETC, as we demonstrated previously^19^.

## Discussion

Several important implications are suggested by our results, all of which help to unify the previously disparate observations about the mechanism of FDXR-based pathogenesis. Most importantly, the results of our iron metabolism assay confirm that FDXR deficiency markedly increases the level of mitochondrial iron in both human and mouse tissues (Fig. 5). This is perhaps not so surprising since iron toxicity has been previously considered as a possible pathogenic contributor to FDXR phenotypes. However, the iron accumulation we have observed here has not been previously reported in the nervous system in the *Fdxr*^*−/−*^ mutant mouse model^21,28^, and provides the first direct evidence that mitochondrial iron accumulation occurs in the nervous system upon *Fdxr* mutation. Total iron levels are also upregulated in the fibroblasts of FDXR patients. Moreover, Prussian blue staining confirms that mitochondrial levels are increased in the brain, liver, heart, and muscle of *Fdxr*^*−/−*^ mutant mice, indicating that a similar pathway is also activated in our mouse model and may contribute to the pathogenic mechanism of FDXR. The fact that iron accumulation is observed in the brain as well as in liver, heart, and muscle tissue also suggests that this pathogenic mechanism may involve both the CNS as well as non-neuronal tissue. This broad pattern of iron accumulation in multiple tissues is reminiscent of the iron accumulation observed in the heart, liver, and spleen of patients with Friedrich’s ataxia^29–31^, further suggesting that FDXR dysfunction may share at least some mechanistic similarities with frataxin dysfunction (perhaps through their shared pathway component FDX2).

Optic atrophy is one of the most striking phenotypes in human patients carrying biallelic FDXR mutations, and our analysis of the *Fdxr*^−/−^ mutant mice only serves to further confirm and clarify this phenomenon. Age-dependent RGC degeneration was confirmed to be the major pathological feature of *Fdxr*-related optic atrophy in mice (Fig. 1). Specifically, our results revealed a significantly reduced thickness in the whole retina and in the ganglion cell complex of adult *Fdxr*^*−/−*^ mutant mice (likely associated with RGC loss and optic nerve degeneration) (Fig. 1), and this loss has been confirmed at the functional level by electroretinography analysis (Fig. 2b). It is unclear if a corresponding loss of photoreceptor cells contributes to this phenotype, as the overall thickness of the outer retina (which contains the photoreceptors) did not appear to be affected. It is possible, however, that photoreceptor function could be compromised without resulting in a loss of the cells themselves. Overall, these results are strikingly similar to the results we observed in our previous findings in *Slc25a46* mutant mice, which showed a similar pattern of optic atrophy and RGC loss to the one that has been observed with *Fdxr*^−/−^ mutants^32,33^. Perhaps one of the most interesting parallels between the *Slc25a46* mice and the *Fdxr*^−/−^ mice is the axonal transport defects observed in the RGCs of *Fdxr*^−/−^ mutants (Fig. 2a), which mirrors the mitochondrial transport defects observed in *Slc25a46*^−/−^ Purkinje cells^33^. It will be interesting to determine if there are other commonalities in the visual impairment phenotype between these two genetic models, or with other more common forms of optic neuropathy such as diabetic retinopathy or open-angle glaucoma.

The optic atrophy phenotype brings up the related issue of neurodegeneration in the FDXR disease model. EMG of the lateral gastrocnemius muscle surrounding the sciatic nerve in adult *Fdxr*^−/−^ mice showed reduced CAMPs and CVs (Fig. 4), which indicates degeneration in the axons of distal sciatic nerves. The reduction of CV in *Fdxr*^−/−^ mutant mice could be secondary to demyelination, which is likely to be the result of axon degeneration induced by compromised mitochondrial function. Our previously published data also show that Fdxr mutation causes inflammation and gliosis in the CNS^20^, and this (combined with iron overload) may cause neurodegeneration and contribute to the pathogenesis of impaired motor coordination in *Fdxr*^−/−^ mice (Fig. 4). It is particularly curious that iron particles are not only deposited inside the neuronal cell bodies, but are also present in the extracellular space (Fig. 5e, f) where they may serve to activate the glial cells that mediate chronic inflammation. How iron accumulation leads to an increase in neurodegeneration has remained unclear until now; however, when combined with our previous report^20^, these results suggest that iron accumulation may enhance inflammation and activation of glia (particularly in the brain), and that this excess inflammation may promote neurodegeneration in *Fdxr* mutants. There is already some precedence for this in mouse models of type I and type II diabetes. These mice, which display retinal degeneration similar to human diabetic retinopathy cases, have also been shown to exhibit both increased iron accumulation and increased inflammatory signals in the retina^34^. It is likely that iron is the agent that induces the inflammation and cell death, as subretinal injections of iron have been previously shown to induce cell death in the RPE layer of the retina via an explicitly inflammatory mechanism^35^. However, there may be other underappreciated factors at play as well. For example, actin aggregation has also been associated with iron accumulation and inflammation in the brain tissue of patients with primary dystonia^36^. On the other hand, it has also been shown that microglia can influence iron accumulation in neurons through secretion of the peptide hormone hepcidin, a process that is itself induced by the neuroinflammation mediator fractalkine^37^. Thus, further study is warranted in all models of neurodegenerative disease to disentangle the complex relationship between inflammation, iron accumulation and cell death, and to clarify any underlying factors that may unite this broad class of disorders.

The dramatic phenotypes caused by *FDXR* mutation shine new light on the importance of the human ferredoxin pathways. Humans have one ferredoxin reductase gene (FDXR)^38^, which is a FAD-dependent enzyme that reduces FDX1 and FDX2 using NADPH as the electron donor^13,16^. Depletion of FDXR diminishes Fe–S cluster assembly and causes mitochondrial iron overload^13^, indicating that FDXR has a crucial role in Fe–S cluster biogenesis in human cells. The surprising number of patient families with FDXR mutations that have been discovered in such a short period of time raises the possibility that the FDXR/FDX1/FDX2 pathway may lie behind many cases of optic atrophy and ataxia with no previously identified cause. In support of this hypothesis, biallelic mutations in the *FDX2* gene (*FDX1L*) have already been identified in patients with metabolic myopathy with deficiencies in various complexes of the ETC, suggestive of mitochondrial Fe–S-related defects^15^. More recently, a case report of individuals from two unrelated Brazilian families carrying the same homozygous mutation in *FDX2* (c.431 C > T, p.P144L) has provided evidence of optic atrophy, myopathy, and axonal neuropathy linked to this variant^39^. Given these facts, we believe that all members of the ferredoxin pathway merit further investigation by the field as a potentially underappreciated source of human mitochondrial disease due to iron misregulation and neurodegeneration.

## Materials and methods

### Mouse strains

All procedures involving mice were approved by either the Institutional Animal Care and Use Committee of Cincinnati Children’s Hospital Medical Center or The Jackson Laboratory’s Institutional Animal Care and Use Committee and performed in accordance with the National Institutes of Health guidelines for the care and use of animals in research. The strain used for this study was B6; 129S-*Fdxr*^*m1J*^ *Otop3*^*m1J*^/GrsrJ (The Jackson Laboratory, Stock #026096). This strain was previously shown to exhibit an abnormal, recessive gait phenotype, and harbors a missense (autosomal recessive) mutation in the *Fdxr* gene resulting in a p.R389Q change in the Fdxr protein^22^. Protein alignment also shows that this amino acid change is located at the same position as mutation R392W in the human ortholog. Although these mice also carry a linked homozygous variant of uncertain clinical significance in the 5′ untranslated region of *Otop3*, it is likely not contributing to the phenotype because mice with a targeted deletion of the *Otop3* gene show increased startle reflex and oligodactyly.

### Cell culture and generation of stable FDXR overexpressing cell line

Fibroblasts derived from patients with *FDXR*^*R392W****/****R392W*^ and *FDXR*^*G443S/F51L*^ genotypes (as well as control fibroblasts) were grown in Dulbecco’s modified Eagle’s medium (DMEM) (Gibco, Thermo Fisher Scientific; Grand Island, NY) supplemented with 10% fetal bovine serum at 37 °C with 5% CO_2_, as described previously^19^. MEFs derived from mice were grown in DMEM (Gibco, Thermo Fisher Scientific; Grand Island, NY) supplemented with 10% fetal bovine serum at 37 °C with 5% CO_2_. WT human *FDXR* cDNA was obtained from Addgene (pTRE-FDXR WT, transcript variant 3, NM_001258012.3) and the stable FDXR overexpressing cell lines were generated, as shown previously^19^.

### Determination of iron levels

Fresh cells or mouse tissue were homogenized in 5× volumes of iron assay buffer using a Dounce homogenizer sitting on ice. Homogenized samples were centrifuged at 16,000×*g* for 10 min, and the supernatant was then collected for the assay (Iron Assay Kit, Abcam, ab83366). The volume of each assay solution was 100 µl (50 µl from each sample combined with 50 µl of the iron assay buffer). In order to determine total iron (II + III) levels, 5 µl of iron reducer was added to each sample, which were then mixed and incubated at 25 °C for 30 min. In total, 100 µl iron probe was added to each well, mixed, and incubated at 25 °C for 60 min while protected from light. At the end of 60 min, the output was recorded immediately on a spectrophotometric microplate reader (OD 593 nm).

### Prussian blue staining

Mice were euthanized in age-matched groups and perfused with 0.9% saline. Frozen tissue sections fixed with 4% PFA were placed in working Iron Stain Solution (Sigma-Aldrich; St. Louis, MO, HT20-1KT). The staining solution consisted of an equal volume of potassium ferrocyanide solution and hydrochloride acid solution. Tissues were stained for 10 min, rinsed in deionized water, and stained in working pararosaniline solution (1 ml of pararosaniline solution added to 50 ml water) for 5 min. The stained tissues were rinsed in deionized water, rapidly dehydrated through alcohol and xylene, and then mounted.

### Mitochondrial membrane potential assay

Human fibroblasts or MEFs at a density of 5 × 10^3^ were plated to each well of a 96-well plate, and cultured in an incubator overnight to allow cell attachment. The next day, the culture medium was aspirated from the plate, and 100 µl of fresh medium was added. For the positive control, CCCP (carbonyl cyanide 3-chlorophenylhydrazone) was added to the control wells for a 50 µM final concentration, and the cells were incubated at 37 °C for 15 min. In total, 10 µl of 2 µM TMRE (tetramethylrhodamine ethyl ester perchlorate) labeling solution was added to each well to get a final concentration of 200 nM, and the plates were placed in an incubator (37 °C, 5% CO_2_) for 20 min. The solution was aspirated from each plate, followed by three washes with warm 1× PBS, and then the addition of 100 µl/well 1× PBS to the plate. Samples were analyzed on a Synergy HTX Multi-Mode Microplate Reader (BioTek Instruments, Inc.; Winooski, Vermont, United States), with excitation at 550 nm and emission at 580 nm (Cell Signaling Technology, #13296).

### Anterograde axonal transport assay in mouse retinal ganglion cells (RGCs)

Seven-month-old B6;129 S *Fdxr*^+/+^ or *Fdxr*^−/−^ mutant mice were anesthetized with an inhalation of 2.5% isoflurane. Using a Hamilton syringe attached with a 33-G needle, 1 µl of 1% cholera toxin subunit B (CTB) conjugated with Alex-Fluor 488 (green) was injected into the left eye of each mouse. The mice were euthanized 2 days later. The mice were then perfused with 4% PFA, and the brains were harvested. The brains were dehydrated in 30% sucrose overnight, and then embedded in OCT. The brains were cryo-sectioned through the superior colliculi with a thickness of 50 µm. The consecutive sections were viewed and imaged under a fluorescent microscope.

### In vivo imaging using OCT

Mice at 10.5 months of age were anesthetized with a mixture of xylazine (6 mg/kg) and ketamine (100 mg/kg). Pupils were dilated with a topical drop of cyclomydril (Alcon Laboratories, Fort Worth, TX). Two minutes after pupil dilation, lubricating eye drops (Alcon Laboratories) were applied to the cornea. Spectral domain OCT with the guidance of bright-field live fundus imaging was performed using an image-guided OCT system (Micron IV, Phoenix Research Laboratories, Pleasanton, CA) according to the manufacturer’s instructions. The vendor’s image acquisition software was used to generate fundus images and OCT scans. The vendor’s software (InSight) was then used to measure thicknesses of retinal layers (NFL + IPL + INL and outer retinal layer) and entire retinas.

### Histological studies

Mice were euthanized in age-matched groups and perfused with 0.9% saline. Tissues were fixed in 10% formalin, then embedded in paraffin, sectioned, and stained with hematoxylin and eosin (H&E). H&E-stained tissues were analyzed by light microscopy. Images were obtained under light microscopy (BX63; Olympus Corporation; Center Valley, PA).

### Gait analysis

Gait analysis was performed by the Mouse Neurobehavioral Phenotyping core at The Jackson Laboratory using the Cleversys ExerGait treadmill system (CleverSys Inc.) equipped with Treadscan software. Following acclimation, test subjects were placed into the treadmill chamber, and the treadmill was slowly increased to 16.7 cm/s. A highspeed camera positioned below the treadmill belt facing the ventral surface (belly) recorded activity of paw placement over an ~5 min duration. Gait measures were defined by the software parameters (www.cleversysinc.com). For analysis, the data for left and right paws were averaged for front and hindpaw gait dynamics. A total of 48 mice (8 mice of each sex/genotype) were tested in three cohorts at two time points, 4–9 weeks of age and 10–13 weeks of age. All technicians were blinded to genotype during testing and analysis. Mice that failed to maintain gait at 16.7 cm/s were excluded.

### Electroretinography (ERG)

A total of 41 mice (at least 6 mice per sex per genotype) were evaluated by electroretinography at 4 months of age. All technicians were blinded to genotype during testing. Following a 2–5 h dark adaptation, mice were anesthetized with an intraperitoneal injection of xylazine (80 mg/kg) and ketamine (16 mg/kg) in normal saline. Dilation of pupils was achieved with one drop of Atropine and 1% Cyclomydril. Rod-mediated ERGs were recorded with the responses to short-wavelength flashes over 4.0-log units to the maximum intensity by a photopic stimulator. Cone-mediated ERGs were recorded with white flashes after 8 min of complete light adaptation. The signals were sampled at 0.8-ms intervals, and were reported as averages^40^.

### Compound muscle action potential (CMAP) recording

Mice were first anesthetized with an intraperitoneal injection of 50 mg/kg sodium phenobarbital. The lateral gastrocnemius muscle was then exposed from the knee to about 4 mm above the ankle. The sciatic nerve was exposed near the biceps femoris muscle. Mylar-coated steel recording wires (California Fine Wire) were implanted into the lateral gastrocnemius muscles, and reference wires were inserted under the skin near the base of the tail. A concentric bipolar stimulating electrode was placed on the sciatic nerve and used for electrical activation.

CMAPs were amplified, recorded with a Micro 1401 data acquisition unit, and analyzed offline with Spike2 software (Cambridge Electronic Design, Cambridge, UK). A 2- to 4-mA electrical stimulation of the sciatic nerve immediately proximal to the tibial, sural, and common peroneal branches was employed via a stimulus isolation unit (WPI) connected to the Micro 1401. After recording, the sciatic nerve was axotomized. The proximal end of the sciatic nerve was stimulated to ensure that CMAPs were generated from direct nerve stimulation. CMAP CV, amplitude, and duration were calculated from each stimulation paradigm. The average stimulation of the sciatic nerve for each paradigm was obtained and averaged across animals.

### Statistical analysis

Results are presented as mean values ± SD or SEM. Graphical illustrations and significance were obtained with GraphPad Prism (GraphPad Software, San Diego, CA, USA) using Student’s *t* test or multiple comparison ANOVA, followed by Bonferroni or Dunnett post hoc tests (according to the sample sets), unless otherwise stated. *P* < 0.05 was considered statistically significant (*P < 0.05; **P < 0.01; ***P < 0.001).). For all statistical tests, the data were confirmed to meet all of the relevant assumptions of the test, including normal distribution and homoscedasticity. The sample sizes were chosen to ensure sufficient statistical power based on previous experience in the lab. No method of randomization was used to determine how samples/animals were allocated to experimental groups.

## Supplementary information

Supplemental Figure S1

Supplemental Figure S2

## References

[1] Wang J, Pantopoulos K (2011). Regulation of cellular iron metabolism. Biochemical J..

[2] Campuzano V (1996). Friedreich’s ataxia: autosomal recessive disease caused by an intronic GAA triplet repeat expansion. Science.

[3] Rotig A (1997). Aconitase and mitochondrial iron-sulphur protein deficiency in Friedreich ataxia. Nat. Genet..

[4] Cavadini P, O’Neill HA, Benada O, Isaya G (2002). Assembly and iron-binding properties of human frataxin, the protein deficient in Friedreich ataxia. Hum. Mol. Genet..

[5] Lodi R (1999). Deficit of in vivo mitochondrial ATP production in patients with Friedreich ataxia. Proc. Natl Acad. Sci. USA.

[6] Durr A (1996). Clinical and genetic abnormalities in patients with Friedreich’s ataxia. N. Engl. J. Med..

[7] Solbach K (2014). Cerebellar pathology in Friedreich’s ataxia: atrophied dentate nuclei with normal iron content. NeuroImage: Clin..

[8] Oakley AE (2007). Individual dopaminergic neurons show raised iron levels in Parkinson disease. Neurology.

[9] Kruer MC (2010). Defective FA2H leads to a novel form of neurodegeneration with brain iron accumulation (NBIA). Ann. Neurol..

[10] Meyer E, Kurian MA, Hayflick SJ (2015). Neurodegeneration with brain iron accumulation: genetic diversity and pathophysiological mechanisms. Annu. Rev. genomics Hum. Genet..

[11] Hayflick SJ (2013). beta-Propeller protein-associated neurodegeneration: a new X-linked dominant disorder with brain iron accumulation. Brain: J. Neurol..

[12] Zhou B (2001). hGFRalpha-4: a new member of the GDNF receptor family and a candidate for NBIA. Pediatr. Neurol..

[13] Shi Y, Ghosh M, Kovtunovych G, Crooks DR, Rouault TA (2012). Both human ferredoxins 1 and 2 and ferredoxin reductase are important for iron-sulfur cluster biogenesis. Biochim. Biophys. Acta.

[14] Sheftel AD (2010). Humans possess two mitochondrial ferredoxins, Fdx1 and Fdx2, with distinct roles in steroidogenesis, heme, and Fe/S cluster biosynthesis. Proc. Natl Acad. Sci. USA.

[15] Spiegel R (2014). Deleterious mutation in FDX1L gene is associated with a novel mitochondrial muscle myopathy. Eur. J. Hum. Genet..

[16] Webert H (2014). Functional reconstitution of mitochondrial Fe/S cluster synthesis on Isu1 reveals the involvement of ferredoxin. Nat. Commun..

[17] Vickery LE (1997). Molecular recognition and electron transfer in mitochondrial steroid hydroxylase systems. Steroids.

[18] Griffin A (2016). Ferredoxin 1b (Fdx1b) is the essential mitochondrial redox partner for cortisol biosynthesis in Zebrafish. Endocrinology.

[19] Peng Y (2017). Biallelic mutations in the ferredoxin reductase gene cause novel mitochondriopathy with optic atrophy. Hum. Mol. Genet..

[20] Slone J (2018). Biallelic mutations in FDXR cause neurodegeneration associated with inflammation. J. Hum. Genet..

[21] Paul A (2017). FDXR mutations cause sensorial neuropathies and expand the spectrum of mitochondrial Fe-S-synthesis diseases. Am. J. Hum. Genet..

[22] Fairfield H (2015). Exome sequencing reveals pathogenic mutations in 91 strains of mice with Mendelian disorders. Genome Res..

[23] Hasegawa T, Ueda T, Okamoto M, Ogata N (2014). Presence of foveal bulge in optical coherence tomographic images in eyes with macular edema associated with branch retinal vein occlusion. Am. J. Ophthalmol..

[24] Semba K (2014). Renin-angiotensin system regulates neurodegeneration in a mouse model of normal tension glaucoma. Cell Death Dis..

[25] Scheld M (2016). Neurodegeneration triggers peripheral immune cell recruitment into the forebrain. J. Neurosci..

[26] Abeti R (2016). ‘Mitochondrial energy imbalance and lipid peroxidation cause cell death in Friedreich’s ataxia’. Cell Death Dis..

[27] Sripetchwandee J, KenKnight SB, Sanit J, Chattipakorn S, Chattipakorn N (2014). Blockade of mitochondrial calcium uniporter prevents cardiac mitochondrial dysfunction caused by iron overload. Acta Physiologica.

[28] Zhang, Y. et al. Ferredoxin reductase is critical for p53-dependent tumor suppression via iron regulatory protein 2. *Genes Dev.*10.1101/gad.299388.117 (2017). 10.1101/gad.299388.117 PMC5558926 28747430

[29] Koeppen AH (2015). The pathogenesis of cardiomyopathy in Friedreich ataxia. PLoS ONE.

[30] Bradley JL (2000). Clinical, biochemical and molecular genetic correlations in Friedreich’s ataxia. Hum. Mol. Genet..

[31] Ramirez RL, Qian J, Santambrogio P, Levi S, Koeppen AH (2012). Relation of cytosolic iron excess to cardiomyopathy of Friedreich’s ataxia. Am. J. Cardiol..

[32] Abrams AJ (2015). Mutations in SLC25A46, encoding a UGO1-like protein, cause an optic atrophy spectrum disorder. Nat. Genet..

[33] Li Z (2017). Loss of SLC25A46 causes neurodegeneration by affecting mitochondrial dynamics and energy production in mice. Hum. Mol. Genet..

[34] Chaudhary K (2018). Iron overload accelerates the progression of diabetic retinopathy in association with increased retinal renin expression. Sci. Rep..

[35] Gelfand BD (2015). Iron toxicity in the retina requires Alu RNA and the NLRP3 inflammasome. Cell Rep..

[36] Gearing M (2002). Aggregation of actin and cofilin in identical twins with juvenile-onset dystonia. Ann. Neurol..

[37] Pandur E (2019). Fractalkine induces hepcidin expression of BV-2 microglia and causes iron accumulation in SH-SY5Y cells. Cell. Mol. Neurobiol..

[38] Solish SB (1988). Human adrenodoxin reductase: two mRNAs encoded by a single gene on chromosome 17cen-q25 are expressed in steroidogenic tissues. Proc. Natl Acad. Sci. USA.

[39] Gurgel-Giannetti J (2018). A novel complex neurological phenotype due to a homozygous mutation in FDX2. Brain: J. Neurol..

[40] Chang B (2007). Two mouse retinal degenerations caused by missense mutations in the beta-subunit of rod cGMP phosphodiesterase gene. Vis. Res..

