# Supplementary figures and images for: Integrated analysis of the molecular pathogenesis of FDXR-associated disease
Source: Cell Death Dis. 2020 Jun 4;11(6):423. doi: 10.1038/s41419-020-2637-3 (PMC7272433; doi:10.1038/s41419-020-2637-3)

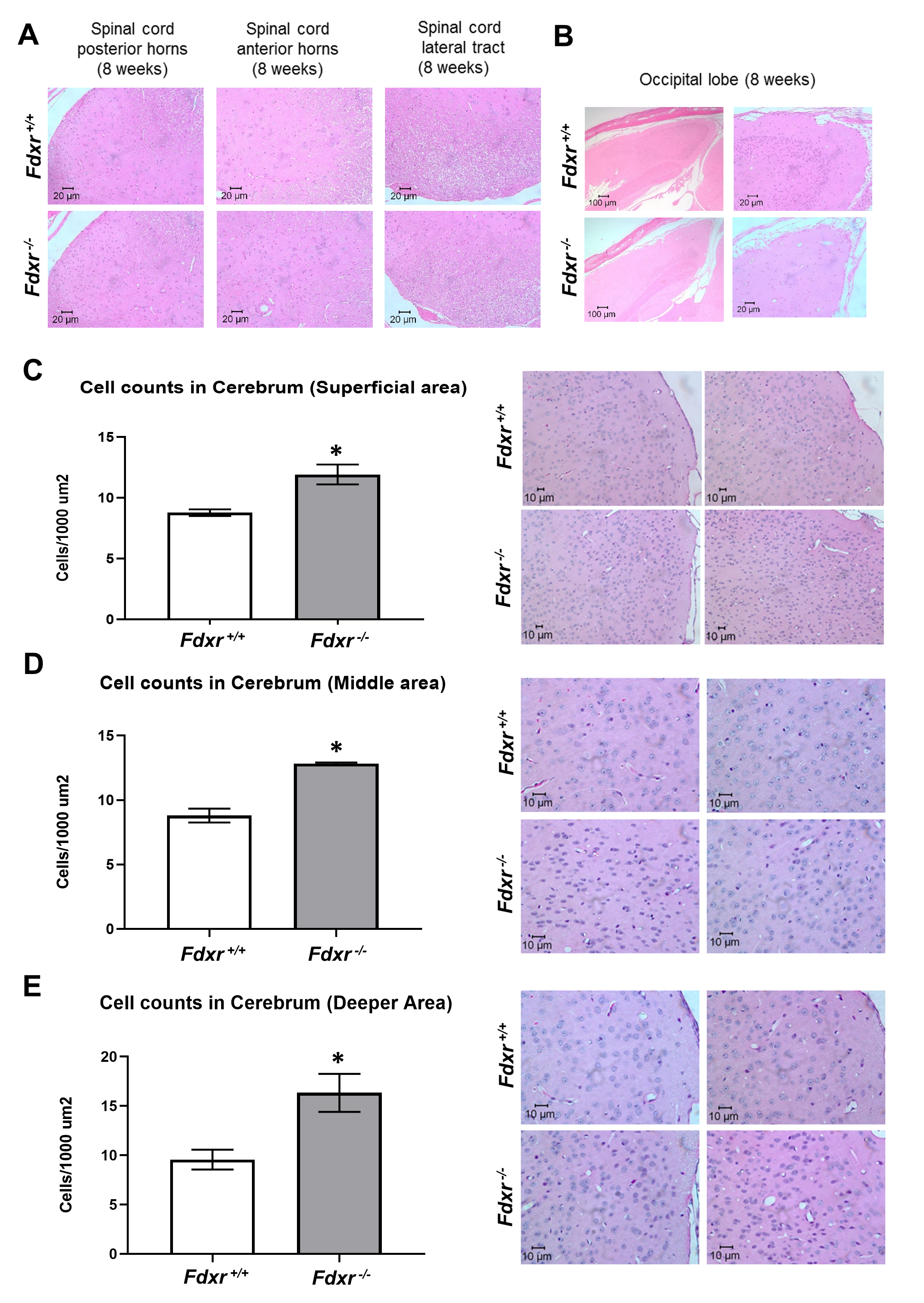

Supplement: Supplementary file 1 — Supplemental Figure S1 [file 41419_2020_2637_MOESM1_ESM.tif]

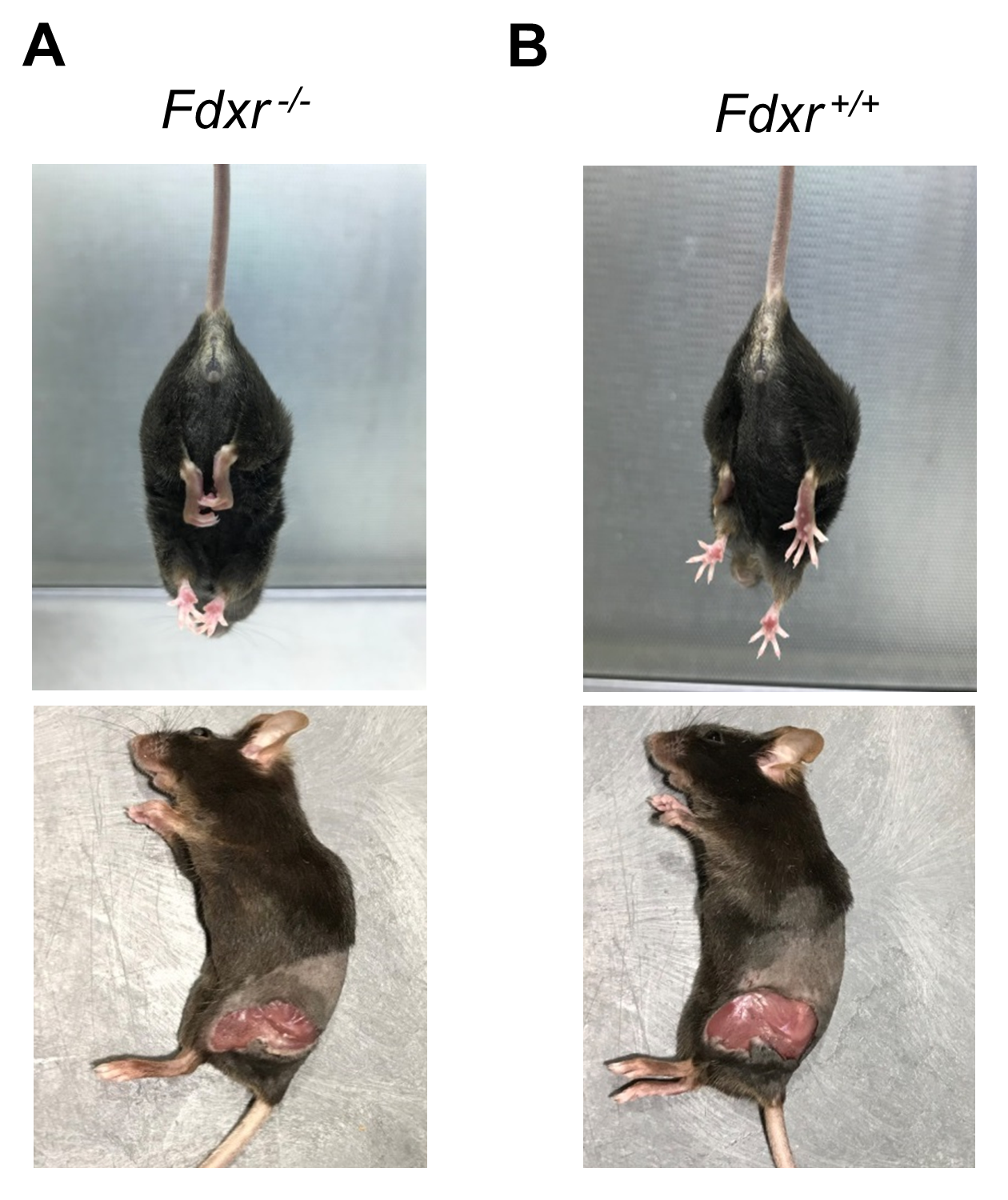

Supplement: Supplementary file 2 — Supplemental Figure S2 [file 41419_2020_2637_MOESM2_ESM.tif]
